# Metal-Based Anticancer Drugs: Integrating Pharmaceutical Perspectives with DFT-Based Quantum Chemical Topology (QTAIM and ELF)

**DOI:** 10.3390/ijms27219748

**Published:** 2026-10-30

**Authors:** Farah Al-Sahlawi, Ali Al-Samydai, Ahlam Hussein Hassan, Amal Mayyas, Mayyas Al-Remawi, Hayder Abdulhussein Shakir, M. R. Mozafari, Marwan Abdelmahmoud Abdelkarim Maki

**Affiliations:** 1 Department of Pharmaceutics, College of Pharmacy, University of Alkafeel, Najaf 61001, Iraq; ahlam.hussein@alkafeel.edu.iq (A.H.H.); hayderalsahlowe@gmail.com (H.A.S.); 2 Al-Kafeel Center for Medical and Pharmaceutical Research, University of Alkafeel, Najaf 61001, Iraq; 3 Pharmacological and Diagnostic Research Centre, Faculty of Pharmacy, Al-Ahliyya Amman University, Amman 19111, Jordan; a.alsamydai@ammanu.edu.jo; 4 Faculty of Health Sciences, American University of Madaba, Madaba 11821, Jordan; a.mayyas@aum.edu.jo; 5 Faculty of Pharmacy and Medical Sciences, University of Petra, Amman 11196, Jordan; malremawi@uop.edu.jo; 6 Australasian Nanoscience and Nanotechnology Initiative (ANNI), Monash University, Clayton, VIC 3800, Australia; 7 Faculty of Pharmaceutical Sciences, UCSI University, Kuala Lumpur 56000, Malaysia

**Keywords:** metal-based anticancer drugs, density functional theory, quantum chemical topology, QTAIM, electron localisation function

## Abstract

Anticancer metal drugs are presented with mechanistically different opportunities in therapeutic use that are not limited by conventional organic chemotherapeutics. Molecular structure not only regulates their clinical behaviour but also their electronic structure, coordination dynamics and metal speciation in complex biological conditions. Rational design of such agents is, nevertheless, a challenging task, even with the long-term interest given, because standard structure–activity relationships are not that effective at revealing such attributes. This review critically studies metal-based anticancer therapeutics in an integrated framework, with a combination of pharmaceutical sciences, density functional theory (DFT), and quantum chemical topology. Following the analysis of significant classes of platinum and non-platinum metal complexes in a structure–reactivity–limitation perspective, the main aspects of pharmacology, such as pharmacokinetics, cellular absorption, resistance, and toxicity, are discussed. The importance of DFT in the explanation of electronic structure is evaluated in addition to the conceptualisations and uses of the Quantum Theory of Atoms in Molecules and the Electron Localisation Function. The focus is on topology-supported relationships between structures and activities that can be integrated into pharmacological results according to electronic and topological measures. Through applications of computational and pharmaceutical knowledge, this review suggests a mechanistically based, multidisciplinary approach to the rational design of next-generation metal-based anticancer drugs.

## 1. Introduction

### 1.1. Rationale for Metal-Based Anticancer Therapeutics

Cancer is one of the major contributors to morbidity and mortality worldwide, placing high pressure on current clinical paradigms with presuppositions that are yet to reach a state of development on molecular grounds [1]. Traditional small-molecule anticancer agents are mostly designed within an organic medicinal chemistry paradigm, wherein shape complementation, noncovalent attractions, and target-directed drug design prevail. Although there have been significant therapeutic achievements using this paradigm per se, it fundamentally limits chemical diversity. It restricts the range of mechanisms that could be used to specifically target malignant cells [2].

Anticancer therapeutics based on metals provide an entirely different chemical and pharmacological explanation. By facilitating the introduction of a metal centre, access to a wide range of different coordination geometries, a variety of different oxidation states, tunable ligand exchange kinetics, and redox activity is facilitated, extending the chemical space of solely organic compounds. These characteristics present new forms of biological interaction that may be unavailable to conventional drugs and enable metal complexes to be used not only as quiescent target binders but also as dynamic chemical systems in response to intracellular signals [3].

The interaction of metal-based agents with DNA, proteins, and redox pathways can be achieved by ligand lability, redox activation, and reactivity by coordination. Notably, these types of properties allow selective activation in tumour microenvironments towards hypoxia, impaired redox status, and altered metal homeostasis. Therefore, metal-based therapeutics are no longer understood as treatments that are limited to the field of oncology [4]. Still, as part of contemporary anticancer approaches, they are promising in overcoming the problem of resistance to drugs, intratumor heterogeneity, and specimen selectivity.

### 1.2. Limitations of Conventional Chemotherapy and the Emergence of Metal Complexes

Systemic toxicity, limited therapeutic indices, and resistance often limit the clinical efficacy of traditional chemotherapeutics. The majority of organic anticancer agents are not selective toward malignant cells, resulting in injury to fast-growing healthy tissues [5]. Additional mechanisms that tend to undermine long-term efficacy are mechanisms of resistance, such as improved DNA repair, metabolic inactivation, elevated efflux, and target mutation [6]. Metal complexes have acquired empirical success within the oncology field, especially those based on platinum, but decades of clinical history have demonstrated their shortcomings, including accumulated toxicity, resistance via detoxification, and uncontrolled biological speciation. These have led to the creation of non-platinum metal complexes and the failure of the organic structure–activity relationship (SAR) to predict metal-based drug behaviour [7].

### 1.3. Need for Integrating Pharmaceutical Sciences with Quantum Chemical Topology

A key challenge in the rational design of metal-based anticancer drugs is connecting pharmaceutical testing and electronic-structure insights. Pharmacokinetics, biodistribution, toxicity, and resistance depend on multiple chemical and biological factors, including metal–ligand bonding, charge distribution, and redox properties [8,9]. Using the Quantum Theory of Atoms in Molecules (QTAIM) and the Electron Localization Function (ELF), respectively, quantum chemical topology offers complementary methods for analysing electron density topology and electron localization [10,11].

Combining these tools with relevant experimental data can support mechanistic interpretation and the development of testable hypotheses for metal-based drug design [9,12,13].

This review highlights the potential benefits of combining pharmaceutical sciences and quantum chemical topology for metal-based anticancer drug development [14]. This strategy may deepen our understanding and help guide the development of a future generation of metal-based therapeutics, for which their efficacy, safety, and translational potential require experimental validation [15].

### 1.4. Literature Search Strategy

A structured literature search was conducted using PubMed/MEDLINE, Scopus, Web of Science, and Google Scholar, focusing on peer-reviewed literature published primarily from 2015 to September 2026. Search terms were used individually and in combination, including metal-based anticancer drugs, platinum, ruthenium, gold complexes, Density Functional Theory (DFT), QTAIM, ELF, SAR, pharmacokinetics, toxicity, and drug resistance. Priority was assigned to original research articles and authoritative reviews that directly addressed the pharmaceutical, biological, and computational aspects of anticancer metal complexes. Older foundational studies were considered to support core concepts. Because the present article is a critical narrative review rather than a systematic review or meta-analysis, study selection was based on relevance to the scope of this review.

## 2. Classes and Structural Diversity of Metal-Based Anticancer Drugs

### 2.1. Platinum-Based Agents: Clinical Successes and Molecular Limitations

Platinum-based drugs are the best-established metal-based anticancer agents that are most clinically validated and still represent a benchmark against which the newer systems are assessed. They are successful because the mechanism of action is relatively well understood and relies on the process of substitution of a ligand to platinum(II) complexes that form covalent adducts with DNA, dominated by coordination to N7 sites of guanine. Such interaction causes intra- and inter-strand crosslinks, bends of the DNA helix, and finally leads to apoptosis. From a coordination chemistry viewpoint, Pt(II) drugs have a square-planar geometry, while their ligand-exchange kinetics, which depend on the ligands and surrounding conditions, influence their stability in the systemic circulation and their activation in the intracellular environment [16]. The evolution of platinum-based anticancer therapy across the first three generations, including representative drugs, their molecular structures, introduction dates, and countries of origin, is summarized in Table 1 [16].

**Table 1:** An overview of key events in the therapeutic use of first-line Pt drugs throughout three generations [16].

Generation	Pt Drug	Molecular Structure	Market Time	Listed Country
First	Cisplatin	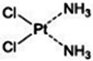	1978	Japan/Italy
Second	Carboplatin	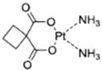	1986	America
Third	Oxaliplatin	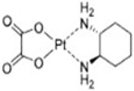	1996	France

**Figure 1:**
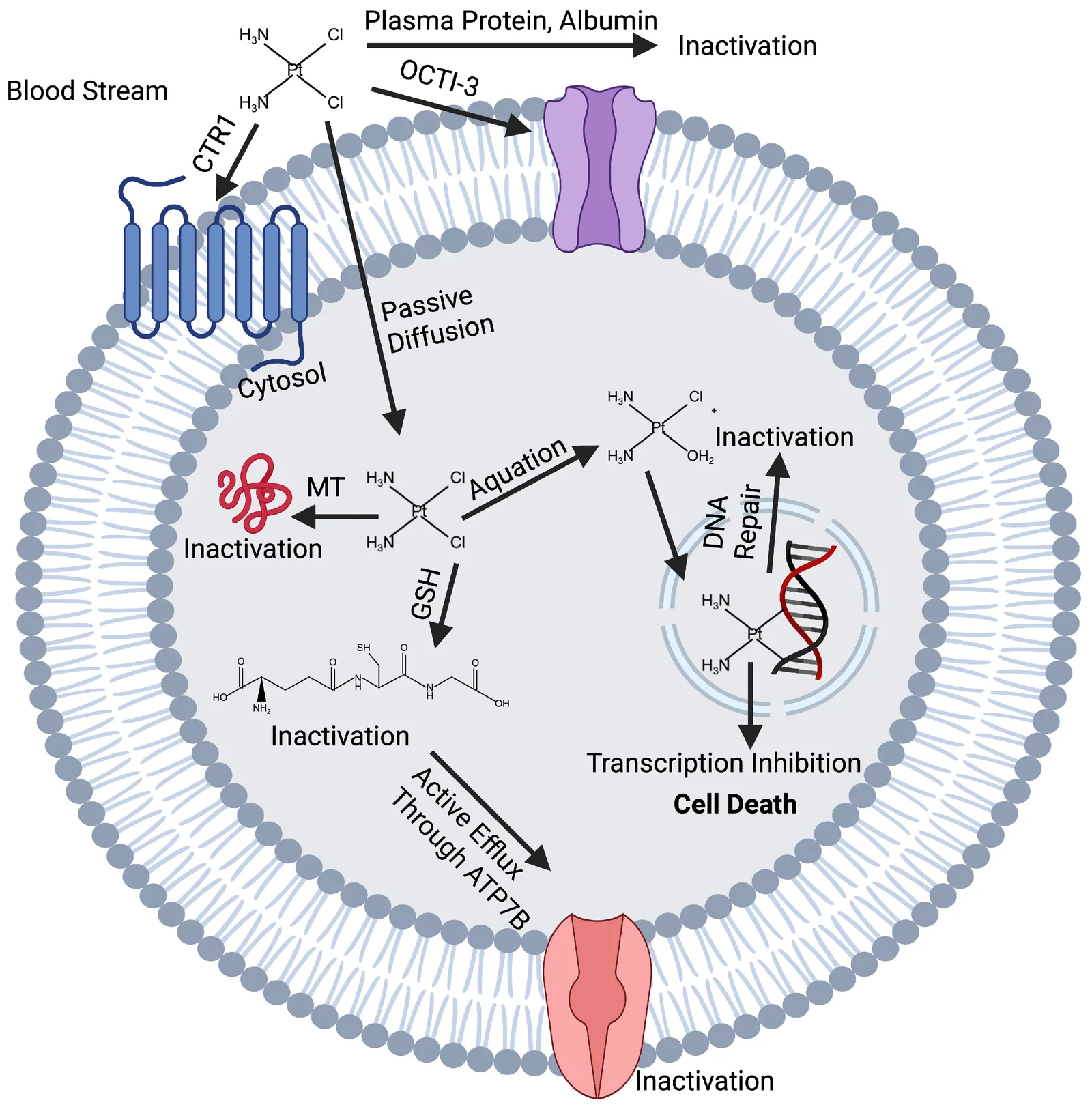
Anticancer mechanism of cisplatin platinum(II). Created by Biorender (https://www.biorender.com).

Although platinum drugs have demonstrated clinical effectiveness, several basic flaws limit their effectiveness for additional therapeutic optimisation. Their mode of action is mostly DNA-based and provides them with minimal flexibility against the changing biological environment of tumours. Mechanisms of resistance include diminished cellular uptake, increased drug efflux, drug inactivation through binding to sulphur-containing biomolecules, and enhanced repair of DNA damage. The processes are closely related to the electronic structure of the platinum complexes, particularly the nature of Pt-ligand bonds and the electrophilicity of the metal centre. Nonetheless, SARs are not in a position to explain why small ligand alterations tend to generate a disproportionate occurrence in cytotoxicity or resistance patterns [17]. The anticancer mechanism of cisplatin, including its interaction with DNA and induction of cellular damage, is illustrated in Figure 1.

Also, due to the absence of selectivity in the use of platinum-based drugs, they cause dose-limiting toxicities, such as nephrotoxicity, neurotoxicity, and ototoxicity. Molecularly, such negative requirements are testimony to the failure of first-generation platinum complexes to discriminate between cancerous and normal cells at the electronic reactivity level. Such weaknesses serve to highlight the necessity of alternative metal systems which are able to offer a greater variety of coordination behaviour, redox responsiveness, and reduced sensitivity to targets [18].

### 2.2. Non-Platinum Metal Complexes in Oncology

#### 2.2.1. Ruthenium-Based Compounds

Ruthenium complexes have become the top alternatives to non-platinum in regard to their rich redox chemistry, kinetic versatility, and their capacity to replicate biologically relevant metal ions. Ruthenium, unlike platinum, is available in a variety of oxidation states under physiological conditions that allow prodrug approaches in which the tumour-specific redox, without adjusting the complex, switches the complex to the active form [19]. In terms of structure, ruthenium anticancer agents with high octahedral geometry offer greater ligand diversity compared to their structure and allow fine-tuning of their electronic behaviour.

Ruthenium systems represent a characteristic benefit due to their decreased need for direct DNA crosslinking. Rather, most complexes preferentially engage with proteins, enzymes, or signalling pathways, which is an indication of the change in rigid DNA damage to dynamic biological modulation. Logically, this mechanism’s versatility, however, brings complexity. Ruthenium complexes are very sensitive to ligand exchange kinetics, coordination sphere distortion, and metal–ligand covalency, which dictate the biological fate of complexes [20]. Even small differences in electronics can dramatically change speciation, biodistribution, and intracellular targets, making it difficult to control a drug through predictive design [21]. The problem with a structure–reactivity viewpoint is trying to correlate the octahedral mechanism of coordination with biological results. Conventional descriptions are insufficient for describing the partial covalency and weak interactions governing interactions between ruthenium and biomolecules, and electronic-structure-based studies which transcend oxidation states and the number of ligands are necessary [22].

#### 2.2.2. Gold Complexes as Targeted Anticancer Agents

Gold complexes strike a new mechanistic departure compared to platinum and ruthenium systems. Instead of binding to DNA, a number of gold(I) and gold(III) complexes can act as powerful enzyme inhibitors with thiols and selenols, especially with respect to redox regulation and mitochondrial activity [23]. The coordination structure of gold(I) complexes frequently exhibits a type of linear coordination, whereas the complexes of gold(III) have the square-planar geometry of platinum(II), but with a much different redox behaviour. This effective anticancer activity of gold complexes is strictly connected with their high affinity toward soft donor atoms, which offer high reactivity against cysteine-rich proteins.

This property allows specific disruption of cellular redox homeostasis; however, it also creates concerns about off-target toxicity and off-target instability in physiological conditions. Particular attention should be paid to ligand design because gold(III) complexes can undergo reduction under physiological conditions, which may alter their speciation and biological activity [23,24].

**Figure 2:**
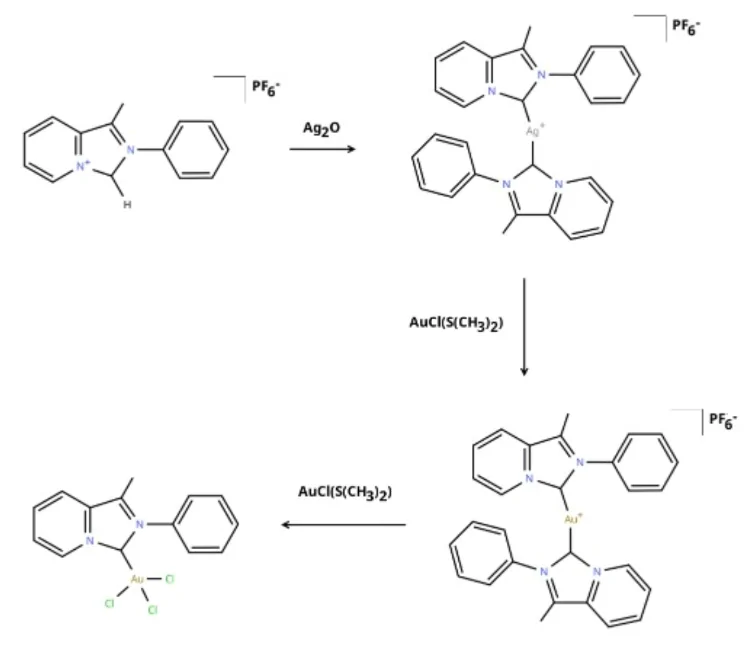
Gold–NHC complexes: from synthetic aspects to anti-cancer activity. Chemical structures were drawn using ChemDraw Professional 26.0 (Revvity Signals Software, Inc., Waltham, MA, USA) [26].

Structurally, gold complexes exemplify how subtleties in the redistribution of electron density and the intensity of the ligand field can predetermine the selectivity and toxicity of a biological agent. The effects are, however, hard to explain through classical coordination chemistry, as they require fine electronic characteristics like the localisation of charge and the character of metal–ligand bonds (Figure 2) [25].

#### 2.2.3. Copper, Iron, and Other Emerging Metal Systems

Complexes made of copper and iron have attracted interest because they are endogenously involved in cellular metabolism and redox. Their biological applicability provides possible benefits in terms of biocompatibility and selective response, especially in tumour environments characterised by high oxidative stress and disturbed metal homeostasis. At the atomic scale, these metals are characterised by adaptable coordination numbers and geometries to allow a variety of ligand assemblies and reactivity schemes [27].

Redox cycling and the generation of reactive oxygen species are often associated with the anticancer effect of copper and iron complexes. Although this mechanism may have a therapeutic benefit, it also poses a great risk of nonselective oxidative injury. As a result, stability and reactivity are among the main design issues. Electronic control should be handled carefully since structural characteristics that increase redox accessibility might also elevate toxicity levels [7]. These novel systems represent a larger problem with respect to non-platinum metal drugs in that they cannot be distinguished by their therapeutic efficacy and electronic structure; at the same time, the relationship between them can hardly be understood using more conventional drug-design-type metrics.

#### 2.2.4. Coordination Chemistry and Structural Diversity of Anticancer Metal Complexes

In all classes of metals, coordination chemistry is the primary determinant of biological behaviour. Stability, reactivity, and target engagement are regulated by geometry, coordination number, ligand denticity, and the associated nature of metal–ligand bonds. In contrast with organic drugs, where the structure is, to a great extent, fixed, metal complexes are dynamic and can change over time in their coordination environments with regard to biological conditions [28]. A variety of processes, including covalent DNA binding, redox modulation, and protein inhibition, are accessible since metal-based anticancer agents possess structural diversity for carrying out these large-scale processes. Nevertheless, this diversity makes rational optimisation more difficult as well. Complexes with similar coordination geometries can exhibit markedly different biological behaviours due to subtle electronic interactions, while pharmacological behaviour may also be independent of coordination geometry [29]. This nonlinearity unveils the defects of conventional structure–activity connections and drives the incorporation of electronic and topological networks to capture bonding characteristics [30].

This, however, is not to view coordination chemistry as a simple structural classification tool but rather as a dynamical scheme that connects electronic structure with pharmacological behaviour. The mechanism of changes in nano-metal–ligand interactions under physiologic conditions remains the subject of initial attempts to develop complexes for which their activity, selectivity, and safety profile are predictable under the influence of physiologic conditions [31].

## 3. Pharmaceutical and Clinical Considerations in Metal-Based Anticancer Therapy

### 3.1. Pharmacokinetics, Biodistribution, and Metal Speciation and Their Clinical Relevance

**Figure 3:**
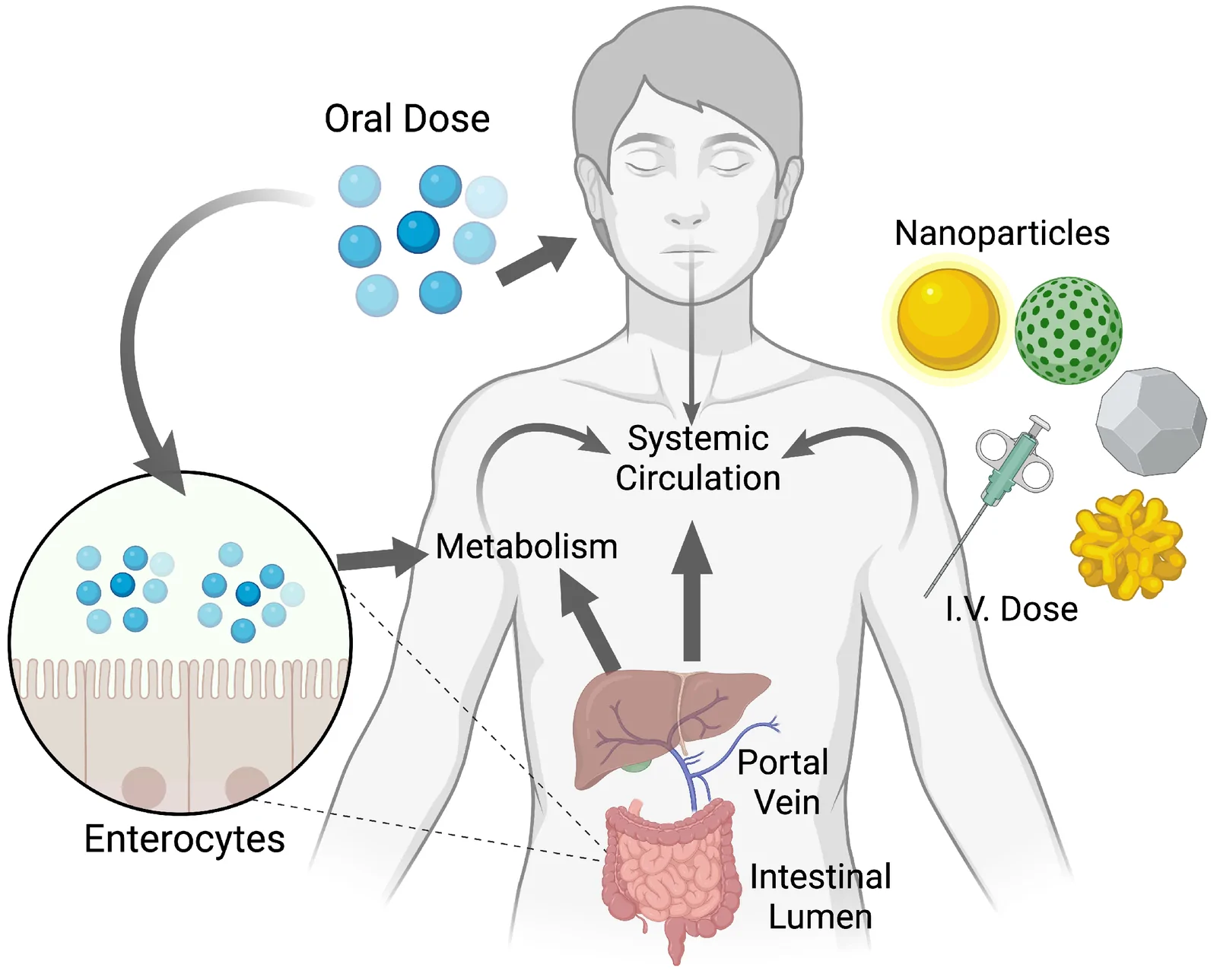
Physiologically based pharmacokinetic and pharmacodynamic models for anticancer nanomedicines. Created by Biorender (https://www.biorender.com).

Pharmacokinetics (PK) is one of the greatest translational application issues of metal-based anticancer drugs, as their in vivo behaviour does not follow the fundamental principles of conventional organic small molecules. The mechanisms of absorption, distribution, metabolism, and excretion of any molecule are not dictated by the mere size and lipophilicity of the molecule but critically depend on the oxidation state of the metal, coordination environment, kinetics of ligand exchange, and dynamic speciation in a physiological environment. Consequently, classical PK models are not very accurate in making predictions of systemic exposure or tissue accumulation of metal complexes [32]. Physiologically based pharmacokinetic and pharmacodynamic (PBPK/PD) models can provide a more comprehensive framework for understanding the absorption, distribution, metabolism, excretion, and therapeutic effects of anticancer nanomedicines (Figure 3).

The process of metal speciation is critical in dictating the biodistribution and therapeutic outcome. When administered, metal-based drugs are subjected to a chemically complex environment that is usually full of competing ligands, i.e., proteins, peptides, and low-molecular-weight thiols. Before the drug reaches the appropriate target, ligand replacement or redox reactions can take place and produce a variety of species with different pharmacological characteristics. These modifications can either allow therapeutic activation, e.g., prodrugs activated by reduction or ligand substitution, or lead to uncontrolled deactivation and toxicity [33]. Notably, speciation cannot be static at any point in time but may be spatially and temporally changing between biological compartments, making the definition of dose–exposure relationships complex.

The biodistributions of metal-based drugs tend to be characterised by robust interactions with plasma proteins and concentration in organs that deal with metal processing, e.g., liver and kidney. Although protein binding can increase circulation times, it can also sequester active species and inhibit tumour delivery. Selective accumulation in tumour tissue, on the other hand, may result from increased permeability and retention effects or metal-specific transport pathways, although these are poorly quantified [34,35]. Clinically, impairment of patient-to-patient variability and a narrow therapeutic window is also associated with the failure to accurately regulate speciation and distribution, which is, in turn, an argument in favour of molecular-level insights that connect electronic structure with pharmacokinetic behaviour [36].

### 3.2. Cellular Uptake, Transporters, and Intracellular Trafficking

The uptake of anticancer metal-based agents in cells has significant discrepancies with that of organic drugs and cannot be reasonably accounted for by passive diffusion. Rather, it is often mediated by a complex synergistic interaction involving active transport, endocytosis, and protein-assisted reactions and is indicative of the chemical similarity of some metal complexes to endogenous metal species. Transporters bound to copper and iron and other metal homeostasis have also been reported to play a role in cellular internalisation of various metal-based drugs; however, their role differs based on coordination geometry, charge, and ligand composition. Intracellular trafficking incorporates a second boundary when it is internalised [37]. The metal complex can either accumulate in a particular organelle or even be involved in a redox reaction or tend to exchange ligands to alter biological activity [38]. As an illustration, chemical degradation or delayed activation may occur due to lysosomal sequestration, or altered cytotoxicity may be caused by mitochondrial accumulation due to redox balance and energy metabolism disruptions. These localisation patterns are not random or accidental, as they are controlled by electronic and structural properties that affect the interactions with biomolecules and membranes [39].

More importantly, accessibility and selectivity of the targets are determined by intracellular trafficking. In order to reach the target of action, metal complexes, which are designed to interact with DNA, proteins, or enzymes, must traverse a crowded intracellular environment in which competitive binding and detoxification routes are endemic. This has been a critical impediment to rational optimisation, with the inability to predict intracellular fate. Pharmacologically, we should learn how the uptake and trafficking of compounds are governed by coordination chemistry and electronic properties in order to design compounds that can be reproduced and for which their behaviour in cells can be learned and controlled [4].

### 3.3. Mechanisms of Drug Resistance and Detoxification Pathways

Resistance to metal-based anticancer drugs involves multiple biological and chemical mechanisms. Lowered cellular internalisation, high cellular efflux, amplified detoxification, and enhanced repair of drug-wrought harm all reduce therapeutic efficacy. In contrast to resistance to organic agents, where they commonly occur by mutation at a certain target cellular metal-binding site or upregulation of a target cellular redox homeostatic mechanism, global adaptations in cellular metal processing and redox homeostasis often cause resistance against metal-based agents [40].

The roles of detoxification pathways are especially important. Biomolecules that contain sulphur, like glutathione and metallothioneins, have the ability to bind metal centres with high affinity, effectively neutralising reactive species and aiding excretion. The tendency of a metal complex to go through such interactions is rigidly connected to its electronic structure, particularly metal electrophilicity and metal–ligand bond polarity. These properties are, however, seldom explicitly included in resistance models, resulting in incomplete mechanistic interpretations [41].

Additional complications when it comes to resistance are efflux transporters that actively eliminate metal complexes or their derivatives within cells. Notably, the mechanisms of resistance might not be consistent between different classes of metals; the approaches that would work with platinum drugs may not necessarily have any effect on redox-active metallic groups like ruthenium or copper complexes [19]. Clinically, resistance is likely to be exhibited in the form of cross-resistance or unresponsive loss, reflecting the failure of existing design paradigms to predict adaptive responses of cells. Resistance must be responded to with a transformation of descriptions of phenomenological forms into mechanistic frameworks that combine electronic reactivity with biological adjustment [42].

### 3.4. Toxicity Profiles and Optimisation of the Therapeutic Index: A Clinical Safety Perspective

The major clinical challenge linked with the development of anticancer agents made of metal is often toxicity, which hinders dose escalation and chronic therapy. Off-target toxicity is not always caused by non-selective target interactions but also by out-of-control chemical reactivity or off-target retention in normal tissues. The closest association between pharmacokinetics and toxicity is particularly obvious in organs that are involved in metal clearance and detoxification [43].

The potential of metal-based drugs to select malignant and normal cells on the basis of molecular distinctions is vital to the therapeutic index of metal-based drugs. Although targeted drug delivery or activation methods could be used to increase tumour selectivity, they are very sensitive to coordination chemistry and electronic stability [8]. High ligand lability or excessive redox reactivity can be very effective in enhancing cytotoxic potential, but at the same time, it augments the number of toxicities in systems. On the contrary, too inert complexes could have desirable safety profiles at the cost of efficacy [44].

Clinically, the safety optimisation of the therapeutic index involves trade-offs between stability, reactivity, and selectivity, and it is challenging to carry out solely using empirical methodologies. Toxicity is closely linked to electronic structure, as metal–ligand bonding, redox behaviour, ligand-exchange kinetics, and chemical reactivity affect both therapeutic and unintended biological interactions. Understanding these molecular and electronic factors is essential for optimising the efficacy, selectivity, and safety of metal-based anticancer agents [45].

## 4. Density Functional Theory (DFT) in Metal-Based Drug Research

### 4.1. Role of DFT in Understanding Electronic Structure, Stability, and Reactivity of Metal-Based Anticancer Agents

DFT has emerged as an essential part of the research on metal-based anticancer agents, as it allows obtaining electronic structure descriptors that regulate the factors of stability, reactivity, and biological effect. Contrary to classical force-field or empirical methods, DFT is explicit in the energy distributions of electron density, making it possible to provide chemically meaningful explanations of bonding between metals and ligands, charge transfer, redox behaviour, etc. This is especially important in cases where multi-electronic states and non-classical bonding are frequently found, such as in transition metal complexes [46].

Within the framework of anticancer drug design, DFT cannot be considered a substitute for biological assays, but it is a mechanistic framework against which the observed pharmacological behaviour can be rationalised. Metal–ligand bond strength, frontier orbital composition, and charge localisation are examples of parameters that provide an understanding of ligand exchange kinetics, redox activation pathways, and the propensity to interact with biomolecular targets. These properties have a direct effect on drug stability during circulation and intracellular activation, and they are also sensitive to detoxification [47].

Nevertheless, the use of DFT depends on attentive interpretation. Calculations of electronic structures are performed on isolated or simplified systems, but the very fabric of biological activity is the operation of grossly complex dynamic environments. The use of single-point descriptors without context validation can easily lead to misleading correlations. Thus, DFT can be considered a hypothesis-generating tool that complements, rather than replaces, experimental pharmacology. It can be better appreciated through the discovery of electronic trends that unveil why structurally related complexes of metals display dissimilar biological behaviour [48].

### 4.2. Selection of Functionals, Basis Sets, and Solvent Models: Practical Considerations and Best Practices

#### 4.2.1. Exchange-Correlation Functionals

The selection of the exchange-correlation functional has been among the most significant decisions in DFT studies of metal-based drugs since its impact directly influences the accuracy of predicted geometries, thermal parameters, and electronic properties [49]. Hybrid functionals like B3LYP offer a balanced approach and are, therefore, computationally efficient. Their propensity to under-represent barriers to reactions and excessive delocalisation of electron density may, however, prove to be problematic in systems involving transition metals, where electron correlations take the centre stage [50].

Functionals with a higher proportion of exact exchange, including PBE0, can give better accounts of the theory of meta–ligand bonds and redox energies, especially those with mixed covalent–ionic interactions [51]. Hybrid Meta-GGA functionals, such as M06, have become popular in bioinorganic applications, as they are parametrised to be more reliable in dealing with transition metals and noncovalent interactions. However, different functional optimisations have no universal optimum, and their differences can be large, particularly with redox-active complexes.

The most important best practice is the comparative application of multiple functional tools to evaluate the strength of electronic trends. The version of functionals promotes confidence in mechanistic interpretations, whereas disagreement indicates the existence of electronic sensitivity that can be biologically useful. Such validation is expected more often by reviewers, especially when the results of DFT are to be used to justify SAR [52].

#### 4.2.2. Basis Sets and Effective Core Potentials

The choice of the basis set is also important in creating a trade-off between computational control and chemical accuracy. In the case of heavy transition metals, very large transition metals are frequently treated with the help of an effective core potential (ECP), e.g., LANL2DZ, to cut down on the costs of computation work by replacing core electrons with pseudopotentials. Although the method allows studying bigger systems, it can impair the accuracy in describing interactions between metals and ligands, as well as charge distributions, unless it is carefully checked [53].

The def2 family provides basis sets of different sizes and includes polarisation functions. These basis sets are all-electron for H–Kr and are used with corresponding effective core potentials for heavier elements; diffuse functions can be added where needed. These basis sets represent a more accurate model of the behaviour of valence electrons, which is necessary in the study of the nature of bonding and reactivity. They, however, have greater computational requirements, which may limit their application to large bioinorganic systems [54].

A practical approach is geometry optimisation with the help of ECP-based basis sets and single-point calculations of energy and electron density with all-electron basis sets of higher quality. This hierarchical analysis provides meaningful electronic analysis with a level of computational feasibility. More importantly, tests of basis set convergence should be mentioned in the case of electronic descriptors in support of biological inferences [53].

#### 4.2.3. Solvent Models and Validation Strategies

Solvent effects play a central role in the behaviour of drugs based on metals, although they are frequently ignored in computations. Implicit solvation models (as polarizable continuum models) are typically employed to model aqueous environments in order to enhance the realism of electronic descriptors. Specific solvent–solute interactions and competitive coordination of biological ligands cannot be described using implicit models, which may have a significant impact on predicted charge distribution, orbital energies, and reaction energetics. Therefore, their application must be seen as a correction of a primary order and not a full representation of biological conditions [55]. In a practical case where competition models of solvent molecules or ligands may provide practical information about the speciation and stability, albeit at a higher computational cost, there should be different experimental measurements, e.g., redox potential, spectroscopy, or accepted reactivity behaviour. Calculations performed by DFT, which are not anchored to experiments, can also be called into question by their reviewer, especially when it comes to interdisciplinary analysis, where it is necessary to prove biological relevance in a convincing manner [56].

### 4.3. Correlating DFT-Derived Descriptors with Biological and Pharmacological Activity

One of the key strengths of density functional theory in metal-based anticancer studies is that it can produce electronic descriptors that can be correlated with biological behaviour. These include the HOMO–LUMO gap, which is commonly used as an electronic descriptor related to chemical reactivity. However, the gap alone does not establish kinetic stability or biological activity, and its interpretation should consider orbital composition and spatial localization [57].

Fukui functions are more mechanistically informative insofar as they allow locating regions of electrophilic or nucleophilic susceptibility, which would allow site-selective reactions with biomolecules to be evaluated. In metal complexes, they differentiate metal-centred and ligand-driven reactivity, which is one of the main aspects when considering mechanisms of action and resistance. However, they are sensitive to the computational setup, which should be carefully interpreted [58].

The interpretation of DNA or protein binding inclinations by examining charge distributions is often done with charge distribution methods, such as partial atomic charges and electron density partitioning. Whereas absolute values are dependent on the method, relative trends in chemically consistent series can be informative when calculated using a consistent methodology. Context-dependent redox descriptors based on DFT can help interpret redox behaviour, although biological redox processes need to be experimentally validated [59].

A computational investigation of 1,2-dihydroquinoline derivatives that integrated quantum–mechanical descriptors with molecular docking and in silico evaluations of bioactivity, ADME properties, and toxicity risks provides an example of a broader pharmaceutical perspective [60]. Although outside the scope of metal-based anticancer drugs, this study highlights the application of electronic descriptors alongside complementary pharmaceutical assessments.

## 5. Quantum Chemical Topology: Conceptual and Theoretical Background

### 5.1. Quantum Theory of Atoms in Molecules (QTAIM): A Framework for Metal–Ligand Bonding

The Quantum Theory of Atoms in Molecules presents an electron-density-based framework within which chemical bonding is analysed, and it especially lends itself to transition metal complexes. Unlike orbital-based bonding analyses, QTAIM is based on the topology of electron density, *ρ(r)*, which can be obtained from quantum–chemical calculations. This concentration is favourable to metal-based anticancer agents, in which bonding frequently leaves classical covalent/ionic boundaries and engages with partial covalency, charge-shift character, and weak interactions that are ill-represented by formal oxidation states or rough ligand field effects [61].

#### 5.1.1. Electron Density Topology and Critical Points

The fundamental principle of QTAIM is the partitioning of molecular space into atomic basins bounded by zero-flux surfaces in the gradient of the electron density. Such partitioning makes atoms in molecules amenable to a chemically reasonable definition, and interatomic interactions are analysed with more than arbitrary assumptions about the nature of bonds [62]. Bond paths and bond critical points (BCPs), which are defined by certain topological properties of *ρ(r)* in any system that bond paths can characterise, are indicators of how atoms interact with each other. Defining the interactions between their basins in the case of metal-based systems, the properties that can be measured at BCPs, including the magnitude of electron density, its Laplacian, and energy density components, can be used to estimate the nature of interactions between atoms and how atoms react. Low electron density and a positive Laplacian at a bond critical point are commonly associated with closed-shell interactions, whereas higher electron density and a negative Laplacian are commonly associated with shared-shell interactions; however, a positive Laplacian does not exclude a covalent contribution in metal–ligand bonds, so energy densities should also be considered [62,63]. Significantly, lots of metal–ligand interactions in biologically significant roles have been found to be in a transitional regime, indicating their mixed bonding nature, which binary labels cannot properly characterise [64].

These topological descriptors can be used in anticancer drug research to obtain systematic comparisons of the metal–ligand bonding of different complexes and ligand environments. This sort of comparison is actually necessary to comprehend fine electronic effects on stability, ligand exchange propensity, and biological deactivation susceptibility. QTAIM is therefore a quantitative language for describing bonding patterns which support pharmacological behaviour [65].

#### 5.1.2. Characterisation of Metal–Ligand Interactions and Biological Relevance

On top of mere bond recognition, QTAIM allows further interrogation of the metal–ligand interactions via combined properties of atomic basins such as atomic charges, atomic volumes, and atomic energies. Such properties are an indication of the distribution of electron density both in the complex and in the response to a change in several systems, such as the coordination environment or oxidation state [61]. In the case of metal-based anticancer agents, basin charges and patterns of charge transfer, in particular, are of particular interest, as they may help interpret differences in the biological activity of structurally similar complexes. Electron density topology variations could give insights into variations in bond polarity or metal electrophilicity that cannot be determined purely on geometry. These insights can be useful in the rationalisation of how subtle ligand changes can result in an inappropriate change in cytotoxicity, resistance, or toxicity [66].

Nevertheless, QTAIM descriptors are not to be regarded independently. The fact that a bond path exists does not mean that it is biologically relevant, and the absence of a BCP does not mean that significant interaction cannot occur under dynamic situations. A strength of QTAIM is that it can give consistent quantitative descriptors, which, when combined with chemical intuition and experimental data, result in a better comprehension of a mechanism. When applied critically, the QTAIM is a strong intermediary between electronic structure and pharmacological results [67].

### 5.2. Electron Localisation Function (ELF): Visualising Electron Pair Distribution

QTAIM analyses the topology of electron density, whereas the Electron Localisation Function complements this approach by providing a measure of electron localisation. ELF has been quite handy in visualising regions of electron localisation that are related to bonding, lone pairs, and core electrons. This physical space image offers intuitive knowledge of chemical bonding, particularly when the use of orbital pictures becomes obscure or inappropriate in metals containing multiple complex systems [68].

#### 5.2.1. Localisation Domains and Chemical Bond Interpretation

ELF splits the molecular space into domains of localisation (that fit into chemically interpretable features), including bonding regions and nonbonding electron pairs. ELF can appear in metal-based anticancer complexes regardless of whether the interaction between the metal and the ligand is dominated by metal-centred electron density, ligand-centred effects, or both, which may signify covalent character. This kind of visualisation can be used to identify strongly localised versus less localised interactions that may influence flexibility and reactivity of the interaction [69]. For example, ELF can be used to identify whether metal–ligand bonds have localised electron pairs to provide qualitative information about bonding patterns. It may also show how electron localisation varies upon redox conversion or ligand swapping, which are fundamental to the biological conversion of metal-based drugs. All these are aspects that cannot be described using standard descriptors and highlight the importance of ELF as a diagnostic instrument [70].

Still, the analysis of ELF interpretations should be based on quantitative ones. Even visualisation can be subjective, and it must be complemented with analogous measures or comparisons of chemically related systems. ELF, when used judiciously, creates more interpretability and does not impinge on rigour.

#### 5.2.2. Complementarity of ELF and QTAIM in Metal-Based Systems

QTAIM used in combination with ELF is more complete and detailed in its description of electronic structure than either treatment by itself. QTAIM is good for measuring interatomic interactions and charge distributions, whereas ELF provides an easy-to-understand illustration of the localisation of electron pairs and bonding patterns. Their combination allows quantitative and qualitative analyses of metal–ligand bonding, which is highly useful in the context of anticancer drug design. QTAIM is able to determine trends in bond character and metal electrophilicity that relate to reactivity and biological interaction, and ELF is able to represent the spatial locations of these trends in a molecule [68]. This combined view makes it easy to interpret the results of DFT in a chemically descriptive language, and it translates electronic descriptors into assumptions about pharmacological behaviour.

## 6. Applications of QTAIM and ELF in Metal-Based Anticancer Drug Design

### 6.1. Metal–Ligand Bonding, Stability, and Reactivity

Among the most effective uses of QTAIM and ELF in the design of metal-based anticancer drugs, explanations of metal–ligand bonding characteristics which regulate biological reactivity and chemical stability should be noted. Drugs with metals should have acceptable stability to enable systemic circulation and regulated reactivity at biological targets [71]. Quantitative evaluation of such a balance is made possible by QTAIM using bond critical point properties, charge distributions, and energy densities relating to the metal and ligand interactions. Differences in these descriptors give information on the lability of a ligand and the probability that the ligand is replaced or activated in a physiological environment [68].

ELF can be used to complete this analysis by localising electron density in metal–ligand bonds. ELF localisation patterns can help characterise bonding, but kinetic inertness and ligand-exchange reactivity require assessments of activation barriers or experimental kinetic data. When combined, QTAIM and ELF will be able to separate complexes that share a common geometry but exhibit strikingly different reactivity profiles in biological media, which provides a mechanistic interpretation of the dramatic pharmacological effect frequently caused by minor changes in ligand structures [63].

### 6.2. Metal–DNA and Metal–Protein Interaction Mechanisms

Interactions between biological agents, such as DNA or proteins, beyond simple covalent bonds mediate the biological activity of many metal-based anticancer agents. QTAIM offers a system used in the identification and characterisation of these interactions, which evaluates electron densities at interaction locations. Coordination bonds, hydrogen bonding, or electrostatic contacts, which stabilise metal–DNA or metal–protein adducts, can be elucidated on bond paths and critical points with weak interactions [72].

QTAIM can help characterise interactions with biomolecular targets, but their persistence and effects on biological function require additional investigation. In proteins, the specific detection of the capacity to bind to sulphur- or nitrogen-containing residues can be achieved, which gives insights into the mechanism of enzyme inhibition or disruption of redox pathways. ELF also indicates these interpretations through visualisation of electron pair localisation changes on biomolecular binding, signalling changes in charge transfer or polarisation of a bond, which form the basis of selectivity and mechanism [73].

### 6.3. Predicting Cytotoxicity, Selectivity, and Anticancer Potency

QTAIM and ELF do not directly predict biological results, but they create descriptors, which are correlated with trends in cytotoxicity and selectivity. The higher electrophilicity of the metals as a result of electron density analysis can lead to better reactivity with respect to nucleophilic cellular targets, which may have better cytotoxic power but also off-target toxicity. Electron density distribution and localisation patterns may help interpret differences in reactivity, but they do not, on their own, establish selective biomolecular interactions. Topological analyses can be used comparatively across systems that are structurally related to identify systemic trends related to favourable therapeutic profiles. Combined with pharmaceutical information, QTAIM and ELF can be used to assist in the informed prioritisation and optimisation of compounds [74].

### 6.4. Representative Computational Applications and Validation Needs

A computational study of cisplatin–carboplatin complexes combined DFT, QTAIM, and molecular docking to examine electronic structure, intermolecular interactions, and possible binding to selected protein targets [71,75]. This provides an example of using electronic and topological analyses alongside docking to formulate hypotheses relevant to metal-based anticancer drug research. However, the reported findings are computational and do not establish improved cytotoxicity, selectivity, pharmacokinetics, or safety. These proposed benefits require experimental validation [76,77].

Notably, there are limitations that are also revealed by such analyses. Electronic topology cannot always explain all biological trends, and there are often discrepancies that can be associated with the effects of the environment, protein dynamics, or adaptive processes in cells. However, the fact that both QTAIM and ELF can be used to explain rather counter-intuitive results highlights their usefulness as predictive tools, both individually and in combination, within an integrated design approach [78].

## 7. Integrating Pharmaceutical Data with Quantum Chemical Topology

### 7.1. Topology-Guided Structure–Activity Relationship (SAR) Development

Traditional SAR models in medicinal chemistry are based largely on organic drug design, in which structural change is correlated with biological reaction by physicochemical measures like lipophilicity, steric effects, and hydrogen-bonding capacity. Although they can be applied effectively to small organic molecules, these models have their own limitations when working with metal-based anticancer agents [79]. Metal complexes are inherently dynamic systems where coordination geometry, oxidation state, and ligand exchange behaviour can change as physiological conditions change. As a result, classical SAR paradigms are not always able to describe counterintuitive trends in nonlinear activity in metal-based drug series [80].

Topology-directed SAR is a non-geometric or non-substituent-directed distillation of geometry-based correlations with electron-density-based interpretations. With the use of QTAIM and ELF descriptors as key variables, the creation of SAR can be developed on electronic properties that directly lead to chemical reactivity and biological interactions. Systematic changes in electron density in metal–ligand-bond critical points can be correlated with ligand lability, redox availability of a ligand, and susceptibility to biomolecular attack, which gives mechanistic explanations of biological trends that would otherwise be empirical [81].

Notably, topology-guided SAR is not aimed at eliminating classical SAR; however, topology-guided SAR is intended to be an extension of classical SAR. Electron-density-based descriptors are valuable complements to conventional SAR as they characterise electronic changes resulting from structural modifications. These descriptors capture variations in atomic and bond properties that are often overlooked by purely geometric or physicochemical descriptors [82]. This method is especially useful with respect to metal complexes, in which small changes in ligands can cause extensive rearrangements of electron density without significant geometric distortion, making it possible to rationalise repeated favourable biological profiles between metal classes.

### 7.2. Translating Electronic and Topological Descriptors into Pharmacological Outcomes

One of the central issues in interdisciplinary drug design is how to translate electronic descriptors into pharmacologically meaningful insights. Quantum chemical topology descriptors can inform the interpretation of chemical reactivity, although these measures need to be contextualised within pharmaceutical outcomes that include efficacy, selectivity, and safety. This requires careful assessment of relationships between electronic features and biological behaviour [83].

Metal electrophilicity assessed from electronic descriptors may help interpret reactivity toward DNA or proteins, but it does not alone predict binding selectivity, cytotoxic potency, or off-target toxicity. Likewise, ELF-derived electron localisation patterns can help interpret bonding changes associated with activation, but they do not alone establish kinetic stability or selective activation in a tumour microenvironment. Such interpretation is best performed through comparative analyses within chemically coherent series rather than absolute thresholds. By incorporating QTAIM and ELF descriptors with experimental PK, cytotoxicity, and resistance data, associations between electronic features and biological outcomes may be explored to help guide rational lead optimisation [82]. Predictive applications require validated quantitative models evaluated against independent experimental data [80,84].

### 7.3. Advantages of an Integrated Multidisciplinary Framework

**Figure 4:**
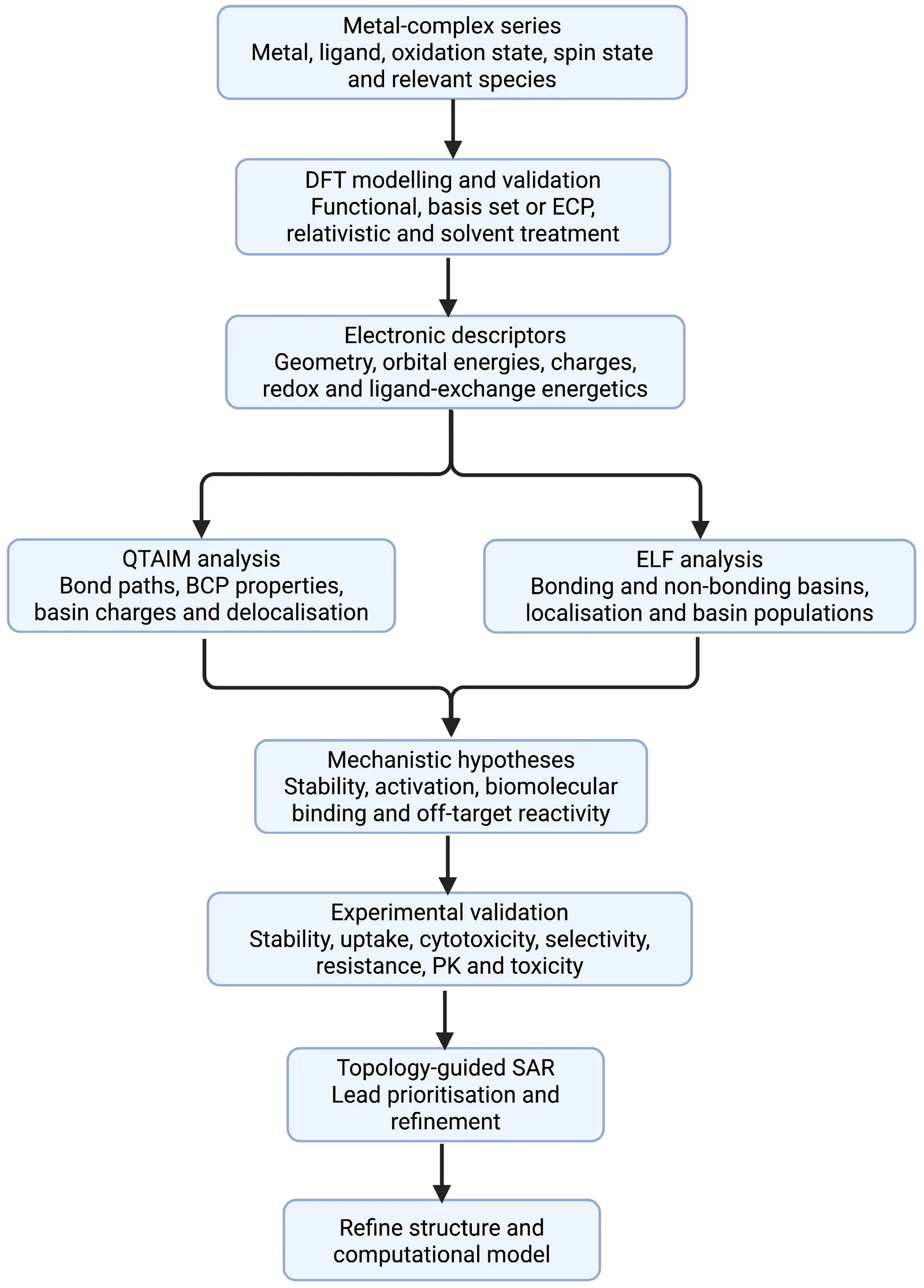
Proposed iterative workflow integrating DFT, QTAIM, ELF, and pharmaceutical data in the design and evaluation of metal-based anticancer agents. Candidate complexes are defined based on their relevant coordination, oxidation, spin, protonation, and speciation states. DFT-derived electronic descriptors and QTAIM/ELF analyses are then translated into hypotheses concerning stability, activation, biomolecular interactions, selectivity, resistance, and toxicity. Experimental pharmaceutical findings are used to validate these hypotheses and guide topology-informed structure–activity relationships and lead optimisation. Created by Biorender (https://www.biorender.com).

The combination of pharmaceutical data and quantum chemical topology has strategic benefits compared to siloed methods. It provides enhanced mechanistic clarification with an explicit association between electron structure and macroscopic drug behaviour, pharmacokinetics and toxicity, and resistance in terms of a metal–ligand bonding continuum and electron density distributions. Second, topology-based insights can help formulate hypotheses about reactivity and guide compound prioritisation for experimental evaluation, while detoxification and biodistribution require additional biological and pharmacokinetic evidence. Third, electronic and topological descriptors are inherent molecular properties, meaning that they can be used to supply a transferable basis for cross-study and cross-metal comparisons, facilitating cumulative knowledge rather than limiting analysis to individual case studies. Together, this combined framework meets more rigorous peer-review demands related to mechanistic depth and gives an easily defensible and logical basis for how metal-based anticancer therapies are designed [85]. To operationalise this integration, we propose the iterative workflow shown in Figure 4. A chemically coherent series of metal complexes is first defined by specifying the metal centre, ligand environment, oxidation state, spin state, coordination geometry, protonation state, and physiologically relevant species. DFT calculations are then performed using appropriately validated functionals, basis sets or effective core potentials, relativistic treatments where required, and suitable solvent models. Conventional electronic descriptors are extracted together with QTAIM properties, including bond paths, bond-critical-point properties, basin charges, delocalisation indices, and ELF properties, including localisation basins and their populations [15].

These descriptors are used to formulate mechanistic hypotheses concerning complex stability, ligand exchange, redox activation, biomolecular binding, and off-target reactivity. The hypotheses are subsequently evaluated against experimental pharmaceutical endpoints, including solution or plasma stability, cellular uptake, cytotoxicity, selectivity, resistance, pharmacokinetics, biodistribution, and toxicity. Agreement or disagreement between the computational and experimental findings guides refinement of both molecular structures and computational models. The proposed framework is therefore comparative and iterative, and it does not treat topological descriptors as direct standalone predictors of clinical efficacy [86].

### 7.4. Novelty Statement and Positioning

Previous studies have utilized electron-density descriptors in drug design [82]; implemented computational approaches for biologically relevant transition-metal complexes [9]; and combined DFT, QTAIM, ELF, and docking analyses for platinum-based systems [71]. Additionally, reviews have examined interactions between anticancer metal complexes and major biological targets [74]. In contrast, the present review uniquely integrates pharmaceutical parameters with DFT, QTAIM, and ELF across various classes of metal-based anticancer agents rather than introducing these methods in isolation.

## 8. Methodological Challenges and Current Limitations: Computational Challenges in Modelling Transition Metal Systems

### 8.1. Computational Challenges in Modelling Transition Metal Systems

Transition-metal complexes present significant challenges for DFT calculations due to closely spaced electronic states, variable spin multiplicities, and pronounced electron correlation. As a result, computed properties are often highly sensitive to the selection of the functional and basis set, underscoring the necessity for rigorous methodological validation [87].

The treatment of heavy metals further enhances these challenges. Relativistic effects, which shape orbital energies and bonding properties, are currently commonly computed using effective core potentials or scalar relativistic corrections. Although they are computationally efficient, these methods may hide delicate electronic characteristics that are important for biological reactivity. Also, the size of biologically relevant metal complexes, especially those that bind large biomolecules, places practical constraints on the accuracy and theory that one can use, and a compromise between accuracy and tractability is necessary [88].

These methodological limitations necessitate interpreting computational results within the context of the specific model employed. While gas-phase calculations or simplified isolated complexes yield valuable insights into intrinsic electronic structure and reactivity, they do not fully capture solvent effects, biomolecular interactions, conformational dynamics, competitive ligand binding, or other aspects of the biological environment. Therefore, electronic-structure descriptors should be considered mechanistic or comparative tools rather than definitive predictors of biological behaviour [9].

### 8.2. Limitations of Quantum Models in Capturing Biological Complexity

Quantum chemical topology and DFT give information on the fine details of electronic structure. Still, they have a fundamental limitation in their applicability to biological systems because of their simplified models. The vast majority of quantum computations are done on isolated complexes or reduced systems, which do not explicitly acknowledge the effects of solvents (or implicitly their dynamics) and cellular heterogeneity. Although this simplification is usually needed, it limits the extent to which pharmacological outcomes can be accurately explained by electronic descriptors [56].

Multiscale processes of biological interactions encompass electronic, molecular, and cellular levels of interaction. Quantum models represent the electronic component well, but these models cannot explain adaptive biological responses, i.e., changes in gene expression, protein turnover, or cellular compartmentalisation. Consequently, relationships between topological descriptors and biological activity are usually indirect and may not generalise beyond closely related chemical series. Moreover, biological targets are dynamic entities. Conformational flexibility of proteins, competitive binding, and post-translational modifications introduce some variability, which is not easily compatible with the study of electronic structures in their tangible form. These constraints do not dilute the importance of quantum chemical topology but emphasise the importance of combining both computational and experimental understanding, as well as biological context [89].

### 8.3. Reproducibility, Validation, and Standardisation Issues

Reproducibility is a major issue in computational studies of metal-based anticancer agents, especially where biological interpretations are intended to be based on electronic descriptors. Inconsistencies due to variations in computational protocols, such as the choice of the functional, choice of the basis set, choice to treat solvation, etc., can make comparisons between studies and across studies difficult. Without standardised methodologies, the generalisability of reported correlations is difficult to evaluate [9].

Electronic descriptors should be validated against experimental data (essentially, but not necessarily, in a systematic manner). Electronic descriptors are believable when benchmarked against measurable properties like redox potentials and spectroscopic signatures or when they exhibit known reactivity trends. However, incomplete reporting of computational methods or selective presentation of favourable results undermines confidence and raises scepticism during peer review [90].

The best practices for making computational parameters more reliable would be transparent reporting of computational parameters, justification of methodological decisions, and sensitivity analyses wherever possible. Creating standards within research communities concerning the use of QTAIM and ELF in bioorganic settings would improve reproducibility and facilitate cumulative advancement even more. The recognition of such limitations is directly related to the enhancement of the validity of integrative strategies. It encourages the acceptance of the latter as complementary, but not definitive, instruments in the research of anticancer drugs [91].

## 9. Future Perspectives and Emerging Trends

### 9.1. AI-Assisted DFT and Topological Analysis in Drug Discovery

Real-time adoption of artificial intelligence (AI) and quantum chemistry is likely to accelerate the discovery of anticancer drugs based on metals. Trained machine learning models that rely on descriptors based on both DFT and topology can trace non-intuitive electronic trends that are associated with chemical stability, reactivity, and biological activity. Unlike all-data models, AI-assisted models, including QTAIM and ELF descriptors, are chemically interpretable and support informed decision-making instead of black-box predictions. These hybrid methods are especially useful in the case of transition metals with large chemical spaces where a trial-and-error approach is not efficient [92].

### 9.2. Toward Personalised Metal-Based Anticancer Therapeutics

Personalisation is a new perspective in oncology using metal-based therapy because it is recognised that the metabolic microsatellite of tissues has significant disparities with respect to redox conditions, homeostasis of metals, and homeostasis of detoxification. Quantum chemical topology descriptors of electronic structures provide a tool for rational correlation of metal complexes with certain biological situations. Through selective redox activity, ligand lability, or metal electrophilicity according to patient-specific tumour features, it might be possible to achieve better selectivity and localise systemic toxicity. However, these applications are currently just a dream; the apparent correspondence between electronic flexibility and biological heterogeneity positions metal-based drugs as promising candidates for personalised intervention strategies [93].

### 9.3. Bridging In Silico Design with Experimental and Clinical Translation

One of the most crucial issues for future research is the conversion of information obtained in silico into experimentally and clinically actionable outputs. This will require the integration of computational modelling, chemical synthesis, and biological evaluation. Recurrent workflows that combine DFT and topology with focused experiments will put mechanistic theories to the test and increase prediction accuracy [94]. Notably, electronic-structure-based design principles will need to be validated in biological models in the process of clinical translation. The ability to transfer quantum chemical topology into multidisciplinary lines of development will allow further studies to cease pointing out correlations and instead deliver topographical anticancer therapeutics using readily available materials, specifically metals [95].

## 10. Conclusions

Anticancer drugs based on metals lie at the intersection of coordination chemistry, pharmacology, and oncology, providing opportunities for therapeutic action that standard organic agents cannot exploit. This paper suggests that their biological behaviour is too complex to be dealt with by either empirical or structure-centred solutions. Rather, rational advancement requires an explicit relationship between electronic structure and pharmacological outcomes.

By combining density functional theory with quantum chemical topology, especially QTAIM and ELF analyses, a mechanistically based system for understanding metal–ligand counteraction, reactivity, and interactions among biomolecules can be elucidated. These methods allow rationalisation of stability, activation, resistance, and toxicity as they go beyond traditional descriptors and are squarely founded in first-principles chemistry. Importantly, topological descriptors in combination with data on pharmaceuticals give some insight as to why structurally similar complexes can have significantly different clinical characteristics.

The topological approach that is taken here does not present quantum chemical topology as a purely predictive solution but rather as an important link between calculations and experiments. Its strength lies in rigorous integration, validation, and comparative analysis. In the future, greater selectivity and increased outside–in topology-informed modelling, combined with data-driven and experimentally guided design, may provide a plausible approach to developing more selective, safer, and mechanistically transparent metal-based anticancer drug therapies.

## Data Availability

No new data were created or analysed in this study. Data sharing is not applicable to this article.

## Abbreviations

The following abbreviations are used in this manuscript:

AI	Artificial intelligence
B3LYP	Becke three-parameter Lee–Yang–Parr functional
BCP	Bond critical point
DFT	Density functional theory
DNA	Deoxyribonucleic acid
ECP	Effective core potential
ELF	Electron localisation function
HOMO	Highest occupied molecular orbital
LANL2DZ	Los Alamos National Laboratory 2 double-zeta basis set
LUMO	Lowest unoccupied molecular orbital
M06	Minnesota 2006 density functional
NHC	N-heterocyclic carbene
PBE0	Perdew–Burke–Ernzerhof hybrid functional
PK	Pharmacokinetics
QTAIM	Quantum theory of atoms in molecules
SAR	Structure–activity relationship
